# New approach for the identification of implausible values and outliers in longitudinal childhood anthropometric data

**DOI:** 10.1016/j.annepidem.2018.01.007

**Published:** 2018-03

**Authors:** Joy Shi, Jill Korsiak, Daniel E. Roth

**Affiliations:** a Centre for Global Child Health and SickKids Research Institute, Hospital for Sick Children, Toronto, ON, Canada; b Department of Pediatrics, Hospital for Sick Children and University of Toronto, Toronto, ON, Canada

**Keywords:** Longitudinal growth data, Jackknife residuals, Biologically implausible values, Outliers

## Abstract

**Purpose:**

We aimed to demonstrate the use of jackknife residuals to take advantage of the longitudinal nature of available growth data in assessing potential biologically implausible values and outliers.

**Methods:**

Artificial errors were induced in 5% of length, weight, and head circumference measurements, measured on 1211 participants from the Maternal Vitamin D for Infant Growth (MDIG) trial from birth to 24 months of age. Each child's sex- and age-standardized z-score or raw measurements were regressed as a function of age in child-specific models. Each error responsible for a biologically implausible decrease between a consecutive pair of measurements was identified based on the higher of the two absolute values of jackknife residuals in each pair. In further analyses, outliers were identified as those values beyond fixed cutoffs of the jackknife residuals (e.g., greater than +5 or less than −5 in primary analyses). Kappa, sensitivity, and specificity were calculated over 1000 simulations to assess the ability of the jackknife residual method to detect induced errors and to compare these methods with the use of conditional growth percentiles and conventional cross-sectional methods.

**Results:**

Among the induced errors that resulted in a biologically implausible decrease in measurement between two consecutive values, the jackknife residual method identified the correct value in 84.3%–91.5% of these instances when applied to the sex- and age-standardized z-scores, with kappa values ranging from 0.685 to 0.795. Sensitivity and specificity of the jackknife method were higher than those of the conditional growth percentile method, but specificity was lower than for conventional cross-sectional methods.

**Conclusions:**

Using jackknife residuals provides a simple method to identify biologically implausible values and outliers in longitudinal child growth data sets in which each child contributes at least 4 serial measurements.

## Introduction

Child growth in stature and body dimensions is a continuous and dynamic process that is optimally studied through longitudinal follow-up. As such, many epidemiologic studies are designed to collect repeated anthropometric measures of each child across successive time points to describe growth patterns, identify predictors, and assess associations with later health outcomes [1], [2], [3]. Standardized procedures for the measurement and collection of anthropometry data have been established to maximize data quality [4], [5], and advanced analytical strategies are employed to accommodate the longitudinal nature of the data collected in such studies [6]; similarly, rigorous quality control procedures during data cleaning are warranted to identify outliers and implausible values.

For cross-sectional studies in which each child only contributes one set of measurements, identification of outliers and implausible values is limited to the use of fixed cutoffs that were derived from a reference population, such as those established for the WHO Child Growth Standards [7] or from the observed distributional properties of the study population. However, with serial anthropometric measurements collected in the same individual, the growth trajectory of each individual provides another basis upon which to assess the biological plausibility of any given measurement for that individual. For example, a decrease in length or height between two successive time points is biologically implausible and thus indicates that at least one of the values was incorrectly measured or recorded. However, without additional information, it is often challenging to determine which of the two values induced the implausible trajectory. Many longitudinal studies use only conventional cross-sectional approaches to identify outliers; when the longitudinal nature of the data is taken into consideration, the methods used to identify these implausible values are often not described, or exclusions are made on a case-by-case basis [8].

Yang et al. [9] recently described the application of conditional growth percentiles for systematically identifying outliers and implausible values in longitudinal childhood anthropometric data. In their example, a hierarchical model of serial weight measurements as a function of age was constructed to estimate an individual's weight percentile at time *t*, while conditioning on the individual's weight percentile at time *t*−*1*. This approach implies that the plausibility of a given measurement is solely based on the preceding measurement (and therefore cannot be applied to an individual's first measurement), yet the expected value is contingent on the overall growth trajectory of the study population.

We propose an alternative longitudinal approach to assess for outliers and implausible values using growth trajectories that are fit to each individual's anthropometric data, in which the identification of outliers and implausible values does not depend on the distribution of measurements in the whole study population. In this study, we aimed to use a longitudinal growth data set with artificially induced errors to demonstrate how jackknife residuals of linear models of z-scores or raw growth data as a function of age can be used to identify biologically implausible values and outliers. We further aimed to compare the sensitivity and specificity of jackknife residuals to alternative methods of data cleaning, including conditional growth percentiles and conventional cross-sectional approaches.

## Methods

### Data source

Anthropometric data collected from infants enrolled in the Maternal Vitamin D for Infant Growth (MDIG) trial were used. Data collection including anthropometry is ongoing; therefore, data available up to January 26, 2017 were used for this study. The MDIG trial methods have been previously described [10]. In brief, the MDIG trial is a randomized placebo-controlled dose-ranging trial of vitamin D supplementation during pregnancy and lactation in Dhaka, Bangladesh. Length, weight, and head circumference measurements are scheduled in tri-monthly intervals from birth to 24 months of age, plus an additional measurement taken at 2, 4, 6, or 8 weeks of age chosen through random assignment, with some variability between infants in actual timing of measurement collection. Age- and sex-standardized z-scores for length, weight, and head circumference measurements were generated using a combination of growth references: the Intergrowth-21st Newborn Size standards; the Intergrowth-21st International Postnatal Growth Standards for Preterm Infants; and the World Health Organization (WHO) Child Growth Standards (Supplementary Material Methods). Data cleaning of any natural occurring errors in the data set was not conducted to preclude biasing the results in favor of one method over another.

### Simulation of implausible values and outliers

Simulated outliers and implausible values were randomly generated in the existing data set by deliberate introduction of data errors. In primary analyses, we randomly selected 5% of all encounters for error induction, in which values were randomly shifted upward or downward in relation to the original observed values. The magnitude of the errors (i.e. differences between original and shifted values) followed a normal distribution of mean = 0 with a standard deviation based off of the derived standard deviation of the raw anthropometric measurements in the WHO Multicentre Growth Reference Study that is the basis for the WHO child growth standards [7]. As such, the standard deviation of the errors varied by type of measurement, sex, and age of the infant. In sensitivity analyses, we used error rates of 10% and 15%, and standard deviations of 2- and 3-times the age- and sex-specific standard deviation for the corresponding anthropometric measure. A Monte Carlo approach was used, in which each scenario for the varying error rates and error generation methods was simulated 1000 times.

### Identifying biologically implausible values

Jackknife residuals were applied to identify the incorrect measurement in instances where errors introduced into the anthropometric data set resulted in a biologically implausible decrease in a child's length, weight, or head circumference from one time point to the next. Jackknife (or externally studentized) residuals, *r*_(*-i*)_, are generated from regression residuals, *e*_*i*_, that are scaled by a function of the mean squared error with the *i*^th^ observation deleted, *MSE*_*(-i)*_, and the leverage, *h*_*i*_:

(1) $$r_{\left(-i\right)}=\frac{e_{i}}{\sqrt{MSE_{\left(-i\right)}\left(1-h_{i}\right)}}$$

Jackknife residuals are expected to follow a *t* distribution with (*n–k–2*) degrees of freedom, where *n* is the number of observations and *k* is the number of parameters in the fitted model, thereby giving the distribution a mean of 0 and standard deviation slightly greater than 1. Given that the *k*^*th*^ observation is an outlier, the jackknife residuals of other observations will shrink toward zero due to an overestimation of MSE, whereas the jackknife residual of the *k*^*th*^ observation will not. As such, jackknife residuals respond more strongly to the presence of a single outlier than does the standardized residual [11].

Any decrease in raw length or head circumference measurements was considered to be biologically implausible, whereas a decrease of greater than 15% in the raw measurements for weight was considered biologically implausible. This analysis addressed instances in which an error in the data set could be clearly identified, but the exact time point at which the error occurred was not as easily discerned. Therefore, these analyses were limited to children for whom there was at least one implausible decrease induced in anthropometric measures.

All individuals with a biologically implausible decrease between any two measurement time points were first identified based on the criteria listed previously. Separately for each child, linear regression was used to fit a straight line through the individual's sex- and age-standardized z-score of the corresponding anthropometry measurement as a function of age:

(2) $$Z_{ij}=\beta_{0i}+\beta_{i}\cdot t_{ij}+\varepsilon_{ij}$$

where “*i*” denotes the *i*^th^ individual and “*j*” denotes the *j*^th^ time point. For raw measurements, each individual's measurements were regressed on the square root of age (t^½^) to model a curvilinear relationship in which growth rates vary with age [12]:

(3) $$Y_{ij}=\beta_{0i}+\beta_{i}\cdot t_{ij}^{1/2}+\varepsilon_{ij}$$

Each measurement of a given individual is assessed for adequate fit to the modeled trajectory for that individual using jackknife residuals (Supplementary Material Methods). We compared the absolute values of the jackknife residuals for the two adjacent time points that spanned the interval across which each biologically implausible decrease occurred. For each pair of values, the value with the largest absolute jackknife residual was labeled as the incorrect value, irrespective of the absolute magnitude of the jackknife residual.

Kappa statistics were computed to assess the performance of these approaches in identifying the correct biologically implausible value (i.e., agreement between the classification by the jackknife residual method vs. the true classification of values as induced errors or unmodified measurements). Analyses were restricted to infants for whom four or more measurement time points were available, since the residual method cannot be applied to individuals with fewer than (*k*+*2*) measurements available, where *k* represents the number of parameters in the model (Supplementary Material Methods). In addition, biologically implausible decreases in the original MDIG data set were excluded from this analysis since the time point at which the error occurred is unknown.

### Comparison of sensitivity and specificity across methods for identifying outliers

To assess the sensitivity and specificity of the jackknife residual method for detecting induced errors, analyses were no longer restricted to instances in which there were implausible decreases in size between subsequent time points, although many of the identified errors were expected to overlap with the biologically implausible values identified in the previous analysis. The modeling strategies were identical to those described previously. All measurements with a jackknife residual below −5 or above +5 were considered outliers. Sensitivity analyses were conducted in which cutoffs of ±3 and ±7 were used instead.

The conditional growth percentile method outlined by Yang et al. was also applied. A random-effects model of the raw anthropometric measurement as a function of age was constructed for the whole study population, using a restricted cubic spline with five knots, where knot locations were based on Harrell's recommendations [13]. Conditional percentiles for each measurement were estimated, and measurements which were below −4 SD or above +4 SD were considered outliers, as implemented by Yang et al. [9]. As per the demonstrations provided by Yang et al., conditional growth percentiles were calculated only for raw measurements and not for their corresponding z-scores because one of its strengths is that the method can be applied even when external standards are not available.

Finally, two traditional cross-sectional approaches to identify outliers and biologically implausible values were applied: (1) using the cutoffs for biologically implausible values that were derived from the WHO Child Growth Standards (<−6 SD or >6 SD for LAZ, <−6 SD or >5 SD for WAZ, and <−5 SD or >5 SD for HCAZ) [7] and (2) using cutoffs of 4 SD below or above the observed population average at the given time point.

For each method, the sensitivity and specificity of the approach was assessed, whereby knowledge of which errors were artificially introduced into the data set was considered the “gold standard”. Sensitivities and specificities were calculated for the whole data set, regardless of the limitations of each method with respect to the detection of errors under certain conditions (e.g., residual method cannot be applied to individuals with fewer than 4 measurements; conditional growth percentile method cannot be applied to an individual's first measurement) and regardless of whether a given measurement was a suspected outlier in the original data set. As such, the sensitivity and specificity of any of these methods were not expected to be 100%. Sensitivity analyses also calculated sensitivity and specificity of these approaches after stratifying by time point or by number of measurement points per individual.

## Results

A total of 8868 length measurements, 8883 weight measurements and 8888 head circumference measurements were available from 1211 infants in the MDIG trial (Table 1). Because data collection is still ongoing, there were fewer measurements available at later time points.

**Table 1:** Summary of anthropometric measurements available from the Maternal Vitamin D for Infant Growth (MDIG) trial∗

Measure	Length	Weight	Head circumference
Number of measurements, by age†			
Birth (0–48 h)	828	835	835
Birth (>48 h)	252	251	252
2 to 8 wk	1095	1099	1100
3 mo	1125	1132	1132
6 mo	1131	1132	1133
9 mo	1126	1126	1126
12 mo	1072	1071	1071
15 mo	880	880	880
18 mo	610	609	610
21 mo	443	442	443
24 mo	306	306	306
Total number of measurements	8868	8883	8888
Number of measurements per infant			
Mean ± SD	7.3 ± 2.0	7.3 ± 2.0	7.3 ± 2.0
Median (range)	7 (1, 11)	7 (1, 11)	7 (1, 11)
Number of infants with			
≥1 measurement	1211	1211	1211
≥2 measurements, n (%)	1196 (98.8)	1196 (98.8)	1196 (98.8)
≥4 measurements, n (%)	1165 (96.2)	1166 (96.3)	1166 (96.3)
≥6 measurements, n (%)	1005 (83.0)	1004 (82.9)	1006 (83.1)
≥8 measurements, n (%)	557 (46.0)	557 (46.0)	557 (46.0)

∗ Based on data available up to January 26, 2017.

† Because of variability in the timing of measurements, these ages represent the scheduled visit time and the actual age of infants at their visit range from the midpoints of adjacent categories (e.g., timing of 6 month measurements range from 4.5 to 7.5 months of age).

After inducing errors at a 5% rate, applying the jackknife residual method to either sex- and age-standardized z-scores or raw measurements performed comparably for both length and weight; the induced errors within each pair were correctly identified in an average of 88%–92% of pairs and kappa statistics ranged from 0.760 to 0.795 (Table 2). For head circumference, applying the jackknife residual method to the sex- and age-standardized z-scores performed better than when applied to the raw measurements (Table 2).

**Table 2:** Comparison of using jackknife residuals from linear versus nonlinear models of z-scores or raw growth data, respectively, as a function of age to identify biologically implausible decreases in length, weight, and head circumference measurements over 1000 simulations with an induced error rate of 5%

Model	Number of pairs of adjacent values with a biologically implausible decrease∗, mean ± SD	Percent of pairs in which the error was correctly identified (%), mean ± SD	Kappa statistic†, mean ± SD
Length			
Model 1‡	62.5 ± 7.9	88.2 ± 4.0	0.760 ± 0.081
Model 2§	62.5 ± 7.9	89.6 ± 3.9	0.788 ± 0.080
Weight			
Model 1‡	26.0 ± 5.3	91.5 ± 5.2	0.795 ± 0.127
Model 2§	26.0 ± 5.3	91.2 ± 5.4	0.789 ± 0.129
Head circumference			
Model 1‡	123.3 ± 10.8	84.3 ± 3.1	0.685 ± 0.062
Model 2§	123.3 ± 10.8	73.2 ± 3.9	0.462 ± 0.079

∗ Any decrease in raw length or head circumference measurements were considered to be biologically implausible, whereas a decrease of greater than 15% in the raw measurements for weight were considered biologically implausible.

† Agreement between the jackknife residual method and truth in the classification of induced plausible values.

‡ Linear equation of sex- and age-standardized z-score as a function of age (*Z*_*ij*_ = *β*_*0i*_ + *β*_*i*_*t*_*ij*_ + *ε*_*ij*_)*.*

§ Raw anthropometric measurement as a function of square root age (*Y*_*ij*_ = *β*_*0i*_ + *β*_*i*_*t*_*ij*_^*½*^ + *ε*_*ij*_)*.*

When using the jackknife residuals method to identify any induced error, sensitivities ranged from 10.7% to 14.1% and specificities ranged from 97.4% to 97.6% when applied to sex- and age-standardized z-scores for length, weight, and head circumference (Table 3). Sensitivity estimates were lower when the jackknife residual method was used for raw length, weight, or head circumference measurements, although specificities were similar to the models based on z-scores (Table 3). Alternative methods to identify induced errors in length, weight, and head circumference measurements had much lower sensitivities (Table 3). The conditional growth percentile method had specificities that were slightly lower than the jackknife residual approach, whereas the conventional cross-sectional methods were very insensitive (<1%) but had nearly perfect specificities (>99%) (Table 3).

**Table 3:** Comparison of alternative methods to identify induced errors in length, weight, and head circumference measurements over 1000 simulations with an induced error rate of 5%

Measure	Jackknife residuals (model 1) with >5 or < −5 cutoff∗	Jackknife residuals (model 2) with >5 or < −5 cutoff†	Conditional growth percentile with >4 or < −4 cutoff‡	Recommended cutoffs from the WHO child growth standards§	>4 or < −4 SD from population average
Length					
Sensitivity (%), mean ± SD	11.9 ± 1.5	10.2 ± 1.4	0.2 ± 0.2	0.1 ± 0.1	0.4 ± 0.3
Specificity (%), mean ± SD	97.4 ± 0.1	97.4 ± 0.1	86.2 ± 0.1	100.0 ± 0.0	99.9 ± 0.0
Weight					
Sensitivity (%), mean ± SD	14.1 ± 1.6	9.7 ± 1.4	0.1 ± 0.2	0.9 ± 0.5	0.6 ± 0.3
Specificity (%), mean ± SD	97.4 ± 0.1	98.0 ± 0.1	86.3 ± 0.1	99.9 ± 0.0	99.9 ± 0.0
Head circumference					
Sensitivity (%), mean ± SD	10.7 ± 1.4	4.1 ± 0.9	0.2 ± 0.2	0.4 ± 0.3	0.5 ± 0.3
Specificity (%), mean ± SD	97.6 ± 0.1	98.1 ± 0.1	86.3 ± 0.1	99.8 ± 0.0	99.8 ± 0.0

∗ Linear equation of sex- and age-standardized z-score as a function of age (*Z*_*ij*_ = *β*_*0i*_ + *β*_*i*_*t*_*ij*_ + *ε*_*ij*_)*.*

† Raw anthropometric measurement as a function of square root of age (*Y*_*ij*_ = *β*_*0i*_ + *β*_*i*_*t*_*ij*_^*½*^*+ ε*_*ij*_).

‡ Based on a random effects restricted cubic spline (with 5 knots) model.

§ For LAZ, <−6 SD or >6 SD; for WAZ, <−6 SD or >5 SD; and for HCAZ, <−5 SD or >5 SD [7].

**Fig. 1:**
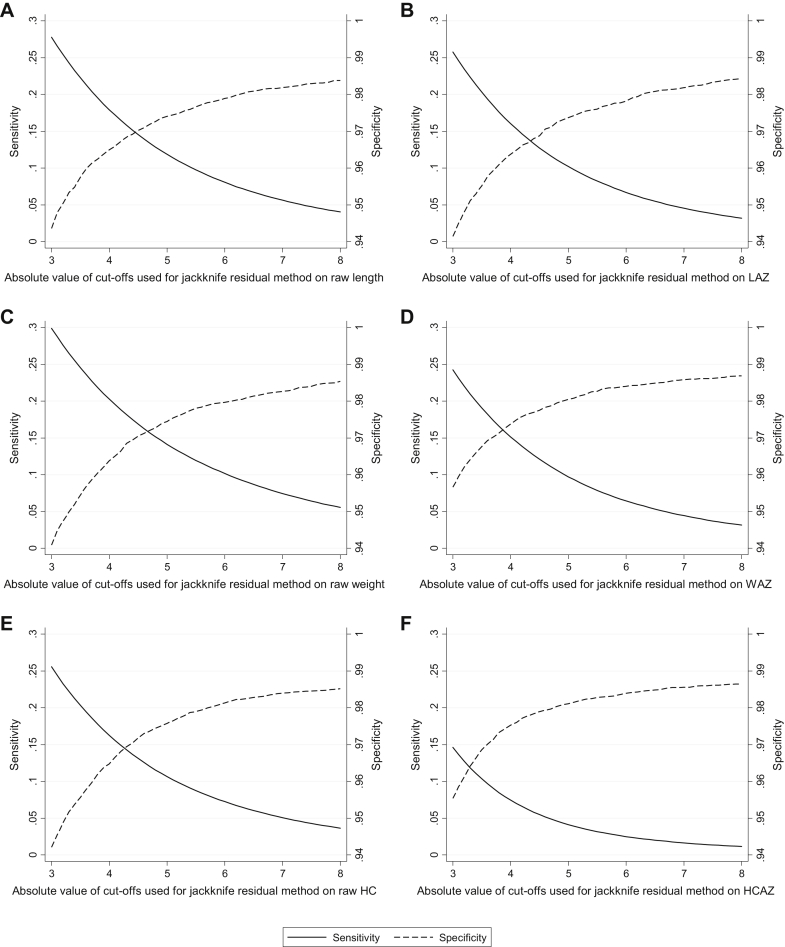
Sensitivity and specificity of the jackknife residual method for detection of outliers in child (A) raw length, (B) length-for-age z-score, (C) raw weight, (D) weight-for-age z-score, (E) raw head circumference, and (F) head circumference-for-age z-score data using cutoffs from ±3 to ± 8.

As expected, sensitivity decreased and specificity increased with increasing absolute values of cutoffs used for the jackknife residual method (Fig. 1, Supplementary Material Table S1). When stratified by the number of measurements available per infant, both the residual method and the conditional growth percentile method had higher sensitivities and lower specificities among participants for whom there were fewer numbers of measurement encounters (Supplementary Material Table S2). When stratified by timing of the measurement, higher sensitivity and lower specificity were also generally observed when the residual method was applied to the first measurement taken for a given individual compared with midtrajectory visits or last visits, although the pattern of differences in sensitivities was not evident for raw measurements (Supplementary Material Table S3). Substantial differences in sensitivity and specificity were not observed between midtrajectory visits and last visits when the conditional growth percentile method was applied. Increasing the overall induced error rate to 10% or 15% resulted in a decrease in sensitivity for the residual method, but specificity was largely unchanged (Supplementary Material Table S4). In contrast, doubling or tripling the width of the distribution of the magnitudes of errors resulted in increased sensitivity, but specificity remained fairly constant (Supplementary Material Table S4). For the alternative methods, changes in the error rate had smaller effects on sensitivities and specificities (Supplementary Material Table S4).

**Fig. 2:**
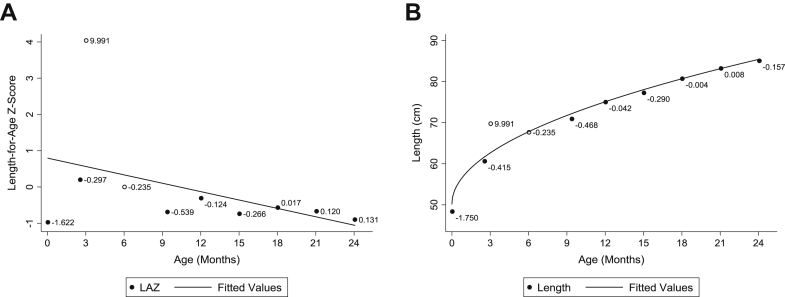
Example of a participant for whom an error was identified within a pair of values in which there was a biologically implausible decrease in length between two adjacent encounters (shown in hollow circles). The error was similarly identified when the jackknife residual method was applied to (A) length-for-age z-scores (LAZ) or (B) raw length measurements. Each measurement is labeled with its corresponding jackknife residual values.

As a case study, the jackknife residual method was applied to the original MDIG data set without induced errors. For length, 21 pairs of measurements with biologically implausible decreases were identified from the data set. An example of an individual with such a pair of measurements is presented in Figure 2, where applying the jackknife residual method to LAZ and raw measurements were consistent in identifying the incorrect measurement time point, as represented by the red marker.

**Fig. 3:**
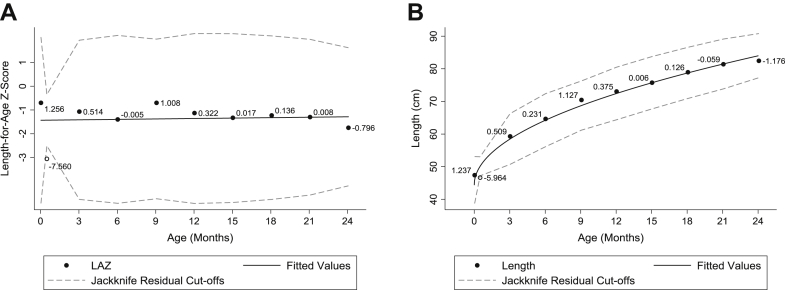
Example of a participant for whom an outlier (shown in hollow circles) was identified when the jackknife residual method is applied to (A) length-for-age z-score (LAZ) or (B) raw length measurement. Each measurement is labeled with its corresponding jackknife residual values.

To reduce the probability of labeling true measurements as errors/outliers by this process, a cutoff of ±5 was used in general, with a more extreme cutoff of ±6 applied to individuals with only 4 to 5 measurements, or if it was an individual's first measurement. As such, of the 8868 available length measurements, the residual method identified 133 (1.50%) outliers when applied to LAZ, and 139 (1.57%) outliers when applied to the raw measurements, with an overlap of 43 (0.48%) measurements that were identified by both methods. A total of 85 (0.96%) measurements could not be evaluated using this method, as these individuals had fewer than 4 separate measurements taken. An example of an outlier identified by both methods is presented in Figure 3.

## Discussion

We demonstrated the use of a novel, simple, and objective approach to identify outliers and biologically implausible values in longitudinal growth data. Although the regression models chosen may provide a crude fit to the data, relative to more sophisticated modeling strategies, this was done intentionally to preclude overfitting the data and biasing the residuals toward the null. The simplicity of the models chosen to assess the jackknife residuals of given measurements will allow this method to be easily applied, especially for large data sets in which manual inspection may not be feasible.

In assessing the application of the jackknife residual method to identify the incorrect measurement within pairs of adjacent values with a biologically implausible decrease, incorrectly labeling an unmodified value as an error or failing to correctly identify an induced error was largely due to instances in which errors were introduced in both measurements within the pair or if the participant had many errors introduced at other measurement time points. As such, the use of the jackknife residual method to identify the incorrect measurement in instances of a biologically implausible decrease is predicated on the assumption that one measurement is correct whereas the other is not—this method is unable to identify instances in which errors occurred at both time points. In addition, increased number and magnitude of errors per individual will distort growth trajectories fit to each individual's measurements and thereby reduce the utility of this approach. Errors in head circumference measurements were more difficult to identify, likely due to the smaller absolute change in head circumference that occurs during development, relative to length and weight. Errors which were large enough to cause a biologically implausible decrease in measurements only caused minor changes in the overall head circumference trajectory, making it difficult for the jackknife residual method, as well as other methods, to discern between correct and incorrect measurements.

The accuracy of the jackknife residual method when applied to age- and sex-standardized z-scores versus raw measurements was comparable, except for head circumference, where using a ±5 cutoff resulted in much higher sensitivity and slightly lower specificity when using HCAZ rather than the raw measurements. Although observed sensitivities may appear low, this was expected since the magnitude of many induced errors were quite small and were very unlikely to be detected using any available method. In addition, since precleaning of the data set was not conducted, naturally occurring errors could be identified as errors and would therefore reduce estimated specificities. However, this would affect not just the jackknife residual method but all methods that were assessed, although likely to different extents. For example, conventional cross-sectional methods have overall lower sensitivity, and therefore are less likely to detect naturally occurring errors. As such, specificity of these methods is less likely to be reduced by naturally occurring errors than for methods which have higher sensitivity such as the jackknife residual method. The effect on estimates of sensitivity are more difficult to predict, but given that the true prevalence of errors in the data set is greater than how much was induced, our estimates of sensitivity may also be an underestimate of the true sensitivity because our sensitivity analyses have shown that the sensitivity of these methods decreased with increasing error rate (Table S4). We also showed that the timing of the measurement as well as the number of measurements available for a given individual has implications for the sensitivity and specificity of the jackknife residual method, but can be accounted for by combining a variety of different cutoffs when flagging potential outliers.

Both sensitivity and specificity were lower when applying the conditional growth percentile method to identify the induced errors compared with the jackknife residual method. The substantially reduced specificity can be attributed to the calculation of specificity in the whole data set, rather than in the subset of measurements to which the method can be applied. For example, the jackknife residual method could not be applied to measurements of individuals who have fewer than four total measurements, which comprised approximately 1% of all measurements in the data set, effectively reducing the observed overall sensitivity of this method by 0.5% and specificity by 0.95% (assuming errors were induced in 5% of those observations, as expected since they were randomly generated). In contrast, the conditional growth percentile method could not be applied to the first measurement time point of each individual, which comprised approximately 13.6% of all measurements in the data set, thus affecting the observed overall sensitivity and specificity of the method to a much greater extent and thereby highlighting the most consequential limitation of this method. The reduced sensitivity of the conditional growth percentile method may be attributed to the use of the 4 SD threshold as recommended by Yang et al [9] to prioritize specificity. Similarly, careful consideration of the sensitivity-specificity trade-off is required for the jackknife residual method, and further inquiry and investigation into outliers flagged by this method should be conducted.

Unsurprisingly, conventional cross-sectional methods performed quite poorly in discriminating between unmodified measurements and induced errors. Although they had near-perfect specificity, a very limited number of the induced errors were detected by these methods, resulting in extremely poor sensitivity. Although lowered cutoffs were assessed to try to increase the sensitivity of these methods (data not shown), substantial increases in sensitivity were not observed until cutoffs were lowered to values that would have eliminated measurements which were likely to be plausible.

Although we demonstrated that jackknife residuals are a practical approach to identify both biologically implausible values and outliers, assumptions were made regarding the functional forms used to reflect the shape of the growth trajectory. Although we used linear equations of z-scores as a function of age or raw anthropometric measurement as a function of square root of age, the general shape of the growth trajectory can be described using other functional forms. Our models were selected on the basis of being generalized forms of the expected growth trajectories of infants from birth to 2 years of age. For example, the linear equation of z-scores as a function of age represents the expected trajectory of a child maintaining a z-score at 0, whereas the square root equation reflects the rapid but decelerating rate of change in raw size measurements that occurs in the postnatal period. Different functional forms may be needed to account for alternate patterns of growth that may be expected with other types or timing of measurements. However, caution is warranted against overfitting the data, as not only will this increase the number of measurements needed per individual, where (*k* + *2*) measurements are needed for a model with *k* parameters, residuals are biased closer toward the null when the data are overfit.

In addition, our simulations assumed an arbitrary error rate and distribution for the magnitude of the errors, which may be impossible to characterize in real longitudinal growth data. However, our sensitivity analyses indicated that although increasing the error rate or magnitude of these errors has implications for the sensitivity of the jackknife residual method, its specificity remains relatively constant. While no single method will be able to identify all errors in a longitudinal growth data set, a combination of approaches, such as applying the jackknife residual method using various regression equations as well as the conditional growth percentile method, may provide the best sensitivity without erroneously identifying real measurements as errors. A multitude of factors, including type of measurement, number of measurements available per individual, and the timing between measurements, should be considered in deciding on a data cleaning strategy. Ultimately, the acceptable balance of sensitivity and specificity—thereby determining the parameters used to implement the method (e.g., cutoff values, use of variable cutoff values for different time points)—is determined by individual investigators based on the study design and overall sample size. Flagged values should undergo manual review and adjudication before being excluded from further analyses; therefore, the choice of cutoffs may be determined by available resources to undertake a manual review process.

In conclusion, the use of jackknife residuals provides a simple and flexible method to identify biologically implausible values and outliers in longitudinal growth data in studies in which most children have at least 4 serial measurements. The detection and correction (or exclusion, if necessary) of measurement errors can increase precision in analyses to identify determinants of growth trajectories or the effects of child growth on later health outcomes.

## Figures and Tables

**Table S1:** Comparison of various cutoffs to identify induced errors in length, weight, and head circumference using the jackknife residual method over 1000 simulations with an induced error rate of 5%

Measure	Jackknife residuals: Model 1∗			Jackknife residuals: Model 2†		
	>3 or < −3 residual cutoff	>5 or < −5 residual cutoff	>7 or < −7 residual cutoff	>3 or < −3 residual cutoff	>5 or < −5 residual cutoff	>7 or < −7 residual cutoff
Length						
Sensitivity (%), mean ± SD	27.8 ± 2.0	11.9 ± 1.5	5.6 ± 1.1	25.8 ± 2.1	10.2 ± 1.4	4.5 ± 1.0
Specificity (%), mean ± SD	94.4 ± 0.1	97.4 ± 0.1	98.2 ± 0.1	94.1 ± 0.1	97.4 ± 0.1	98.2 ± 0.1
Weight						
Sensitivity (%), mean ± SD	29.9 ± 2.1	14.1 ± 1.6	7.4 ± 1.2	24.2 ± 1.9	9.7 ± 1.4	4.4 ± 1.0
Specificity (%), mean ± SD	94.1 ± 0.1	97.4 ± 0.1	98.3 ± 0.1	95.7 ± 0.1	98.0 ± 0.1	98.6 ± 0.0
Head circumference						
Sensitivity (%), mean ± SD	25.6 ± 1.9	10.7 ± 1.4	5.1 ± 1.0	14.6 ± 1.6	4.1 ± 0.9	1.6 ± 0.6
Specificity (%), mean ± SD	94.2 ± 0.1	97.6 ± 0.1	98.4 ± 0.1	95.5 ± 0.1	98.1 ± 0.1	98.6 ± 0.0

∗ Linear equation of sex- and age-standardized z-score as a function of age (*Z*_*ij*_ = *β*_*0i*_ + *β*_*i*_*t*_*ij*_ + *ε*_*ij*_)*.*

† Raw anthropometric measurement as a function of square root of age (*Y*_*ij*_ = *β*_*0i*_ + *β*_*i*_*t*_*ij*_^*½*^ + *ε*_*ij*_)*.*

**Table S2:** Comparison of various methods to identify induced errors in length, weight, and head circumference measurements over 1000 simulations with an induced error rate of 5%, stratified by the number of measurements available

Number of observations per infant	Jackknife residuals (model 1) with >5 or < −5 cutoff∗		Jackknife residuals (model 2) with >5 or < −5 cutoff†		Conditional growth percentile with >4 SD or < −4 SD cutoff‡	
	Sensitivity (%), mean ± SD	Specificity (%), mean ± SD	Sensitivity (%), mean ± SD	Specificity (%), mean ± SD	Sensitivity (%), mean ± SD	Specificity (%), mean ± SD
Length						
2–3 measurements per infant	—	—	—	—	1.6 ± 8.7	54.2 ± 1.4
4−5 measurements per infant	19.0 ± 6.2	92.7 ± 0.4	17.1 ± 6.2	93.1 ± 0.4	0.1 ± 0.6	79.1 ± 0.3
6−7 measurements per infant	13.3 ± 2.7	98.5 ± 0.1	10.7 ± 2.5	98.1 ± 0.1	0.1 ± 0.3	84.6 ± 0.2
≥8 measurements per infant	10.2 ± 1.8	99.1 ± 0.1	9.0 ± 1.7	99.2 ± 0.1	0.2 ± 0.3	88.9 ± 0.1
Overall	11.9 ± 1.5	97.4 ± 0.1	10.2 ± 1.4	97.4 ± 0.1	0.2 ± 0.2	86.2 ± 0.1
Weight						
2−3 measurements per infant	—	—	—	—	0.1 ± 1.7	56.4 ± 1.4
4−5 measurements per infant	21.0 ± 6.3	94.5 ± 0.4	15.7 ± 6.1	95.1 ± 0.4	0.1 ± 0.5	79.3 ± 0.3
6−7 measurements per infant	15.6 ± 3.0	97.9 ± 0.1	10.3 ± 2.5	98.7 ± 0.1	0.1 ± 0.3	84.7 ± 0.2
≥8 measurements per infant	12.4 ± 2.1	99.2 ± 0.1	8.6 ± 1.8	99.8 ± 0.0	0.1 ± 0.2	89.0 ± 0.1
Overall	14.1 ± 1.6	97.4 ± 0.1	9.7 ± 1.4	98.0 ± 0.1	0.1 ± 0.2	86.3 ± 0.1
Head circumference						
2−3 measurements per infant	—	—	—	—	0.2 ± 2.8	57.0 ± 1.4
4−5 measurements per infant	20.0 ± 6.5	95.1 ± 0.4	14.2 ± 5.3	94.9 ± 0.4	0.2 ± 0.7	79.3 ± 0.3
6−7 measurements per infant	12.4 ± 2.7	98.4 ± 0.1	5.2 ± 1.8	99.2 ± 0.1	0.3 ± 0.4	84.6 ± 0.2
≥8 measurements per infant	8.4 ± 1.7	99.1 ± 0.1	2.0 ± 0.9	99.6 ± 0.1	0.3 ± 0.3	88.9 ± 0.1
Overall	10.7 ± 1.4	97.6 ± 0.1	4.1 ± 0.9	98.1 ± 0.1	0.3 ± 0.2	86.3 ± 0.1

∗ Linear equation of sex- and age-standardized z-score as a function of age (*Z*_*ij*_ = *β*_*0i*_ + *β*_*i*_*t*_*ij*_ + *ε*_*ij*_).

† Raw anthropometric measurement as a function of square root of age (*Y*_*ij*_ = *β*_*0i*_ + *β*_*i*_*t*_*ij*_^*½*^ + *ε*_*ij*_)*.*

‡ Based on a random effects restricted cubic spline (with 5 knots) model.

**Table S3:** Comparison of various methods to identify induced errors in length, weight, and head circumference measurements among infants with at least 2 measurements over 1000 simulations with an induced error rate of 5%, stratified by visit at which error was induced

Timing of visit	Jackknife residuals (model 1) with >5 or < −5 cutoff∗		Jackknife residuals (model 2) with >5 or < −5 cutoff†		Conditional growth percentile with >4 SD or < −4 SD cutoff‡	
	Sensitivity (%), mean ± SD	Specificity (%), mean ± SD	Sensitivity (%), mean ± SD	Specificity (%), mean ± SD	Sensitivity (%), mean ± SD	Specificity (%), mean ± SD
Length						
First visit	17.6 ± 4.8	91.7 ± 0.4	11.2 ± 4.1	90.9 ± 0.4	—	—
Middle visit	11.5 ± 1.8	98.9 ± 0.1	9.8 ± 1.7	99.0 ± 0.1	0.2 ± 0.3	99.8 ± 0.0
Last visit	8.1 ± 3.4	96.4 ± 0.2	11.7 ± 4.2	96.5 ± 0.2	0.2 ± 0.5	99.8 ± 0.0
Overall	11.9 ± 1.5	97.4 ± 0.1	10.2 ± 1.4	97.4 ± 0.1	0.2 ± 0.2	86.2 ± 0.1
Weight						
First visit	21.9 ± 5.5	90.0 ± 0.4	5.9 ± 3.0	93.5 ± 0.3	—	—
Middle visit	13.3 ± 1.9	99.2 ± 0.1	9.5 ± 1.6	99.4 ± 0.0	0.2 ± 0.2	99.9 ± 0.0
Last visit	11.1 ± 4.1	96.7 ± 0.2	14.8 ± 4.6	96.6 ± 0.2	0.0 ± 0.2	100.0 ± 0.0
Overall	14.1 ± 1.6	97.4 ± 0.1	9.7 ± 1.4	98.0 ± 0.1	0.1 ± 0.2	86.3 ± 0.1
Head circumference						
First visit	17.5 ± 5.1	91.6 ± 0.4	6.2 ± 3.2	94.6 ± 0.3	—	—
Middle visit	10.2 ± 1.6	99.0 ± 0.1	3.0 ± 0.9	99.5 ± 0.0	0.4 ± 0.3	99.8 ± 0.0
Last visit	6.3 ± 3.1	97.2 ± 0.2	8.1 ± 3.6	95.5 ± 0.3	0.1 ± 0.4	99.9 ± 0.0
Overall	10.7 ± 1.4	97.6 ± 0.1	4.1 ± 0.9	98.1 ± 0.1	0.3 ± 0.2	86.3 ± 0.1

∗ Linear equation of sex- and age-standardized z-score as a function of age (*Z*_*ij*_ = *β*_*0i*_ + *β*_*i*_*t*_*ij*_ + *ε*_*ij*_)*.*

† Raw anthropometric measurement as a function of square root of age (*Y*_*ij*_ = *β*_*0i*_ + *β*_*i*_*t*_*ij*_^*½*^ + *ε*_*ij*_)*.*

‡ Based on a random effects restricted cubic spline (with 5 knots) model.

**Table S4:** Comparison of various methods to identify induced errors in length, weight, and head circumference measurements over 1000 simulations with induced error rates of 5%, 10%, and 15%

Error rate	Jackknife residuals (model 1) with >5 or < −5 cutoff∗		Jackknife residuals (model 2) with >5 or < −5 cutoff†		Conditional growth percentile with >4 SD or < −4 SD cutoff‡		Recommended cutoffs from the WHO child growth standards		>4 or < −4 SD from population average	
	Sensitivity (%), mean ± SD	Specificity (%), mean ± SD	Sensitivity (%), mean ± SD	Specificity (%), mean ± SD	Sensitivity (%), mean ± SD	Specificity (%), mean ± SD	Sensitivity (%), mean ± SD	Specificity (%), mean ± SD	Sensitivity (%), mean ± SD	Specificity (%), mean ± SD
Length										
5% error rate	11.9 ± 1.5	97.4 ± 0.1	10.2 ± 1.4	97.4 ± 0.1	0.2 ± 0.2	86.2 ± 0.1	0.1 ± 0.1	100.0 ± 0.0	0.4 ± 0.3	99.9 ± 0.0
10% error rate	2.3 ± 0.7	96.7 ± 0.1	2.2 ± 0.7	96.8 ± 0.1	0.1 ± 0.2	86.2 ± 0.1	0.1 ± 0.1	100.0 ± 0.0	0.2 ± 0.2	99.9 ± 0.0
15% error rate	2.4 ± 0.7	96.7 ± 0.1	2.3 ± 0.7	96.8 ± 0.1	0.1 ± 0.1	86.2 ± 0.1	0.1 ± 0.1	99.9 ± 0.0	0.1 ± 0.2	99.9 ± 0.0
Weight										
5% error rate	14.1 ± 1.6	97.4 ± 0.1	9.7 ± 1.4	98.0 ± 0.1	0.1 ± 0.2	86.3 ± 0.1	0.2 ± 0.2	99.9 ± 0.0	0.6 ± 0.3	99.9 ± 0.0
10% error rate	2.5 ± 0.8	96.6 ± 0.1	1.6 ± 0.6	97.4 ± 0.1	0.1 ± 0.1	86.3 ± 0.1	0.1 ± 0.1	99.9 ± 0.0	0.1 ± 0.2	99.8 ± 0.0
15% error rate	2.6 ± 0.8	96.5 ± 0.1	1.8 ± 0.7	97.2 ± 0.1	0.1 ± 0.1	86.3 ± 0.1	0.1 ± 0.1	99.9 ± 0.0	0.1 ± 0.2	99.9 ± 0.0
Head circumference										
5% error rate	10.7 ± 1.4	97.6 ± 0.1	4.1 ± 0.9	98.1 ± 0.1	0.3 ± 0.2	86.3 ± 0.1	0.4 ± 0.3	99.8 ± 0.0	0.5 ± 0.3	99.8 ± 0.0
10% error rate	2.1 ± 0.7	97.0 ± 0.1	1.2 ± 0.5	97.9 ± 0.1	0.1 ± 0.2	86.3 ± 0.1	0.2 ± 0.2	99.8 ± 0.0	0.2 ± 0.2	99.8 ± 0.0
15% error rate	2.2 ± 0.7	96.9 ± 0.1	1.3 ± 0.5	97.8 ± 0.1	0.1 ± 0.1	86.3 ± 0.1	0.2 ± 0.2	99.8 ± 0.0	0.2 ± 0.2	99.8 ± 0.0

∗ Linear equation of sex- and age-standardized z-score as a function of age (*Z*_*ij*_ = *β*_*0i*_ + *β*_*i*_*t*_*ij*_ + *ε*_*ij*_)*.*

† Raw anthropometric measurement as a function of square root of age (*Y*_*ij*_ = *β*_*0i*_ + *β*_*i*_*t*_*ij*_^*½*^ + *ε*_*ij*_)*.*

‡ Based on a random effects restricted cubic spline (with 5 knots) model.

**Table S5:** Comparison of various methods to identify induced errors in length, weight, and head circumference measurements over 1000 simulations in which magnitude of induced errors have standard deviations of 1, 2, and 3

	Jackknife residuals (model 1) with >5 or < −5 cutoff∗		Jackknife residuals (model 2) with >5 or < −5 cutoff†		Conditional growth percentile with >4 SD or < −4 SD cutoff‡		Recommended cutoffs from the WHO child growth standards		>4 or < −4 SD from population average	
	Sensitivity (%), mean ± SD	Specificity (%), mean ± SD	Sensitivity (%), mean ± SD	Specificity (%), mean ± SD	Sensitivity (%), mean ± SD	Specificity (%), mean ± SD	Sensitivity (%), mean ± SD	Specificity (%), mean ± SD	Sensitivity (%), mean ± SD	Specificity (%), mean ± SD
Length										
SD 1	11.9 ± 1.5	97.4 ± 0.1	10.2 ± 1.4	97.4 ± 0.1	0.2 ± 0.2	86.2 ± 0.1	0.1 ± 0.1	100.0 ± 0.0	0.4 ± 0.3	99.9 ± 0.0
SD 2	29.3 ± 2.2	97.5 ± 0.1	26.9 ± 2.1	97.5 ± 0.1	2.8 ± 0.7	86.3 ± 0.1	1.4 ± 0.6	100.0 ± 0.0	3.5 ± 0.8	99.9 ± 0.0
SD 3	41.0 ± 2.3	97.5 ± 0.1	38.9 ± 2.3	97.5 ± 0.1	9.1 ± 1.2	86.3 ± 0.1	6.6 ± 1.2	100.0 ± 0.0	10.0 ± 1.2	99.9 ± 0.0
Weight										
SD 1	14.1 ± 1.6	97.4 ± 0.1	9.7 ± 1.4	98.0 ± 0.1	0.1 ± 0.2	86.3 ± 0.1	0.2 ± 0.2	99.9 ± 0.0	0.6 ± 0.3	99.9 ± 0.0
SD 2	30.7 ± 2.2	97.5 ± 0.1	25.2 ± 2.1	98.1 ± 0.1	3.1 ± 0.8	86.3 ± 0.1	3.8 ± 0.9	99.9 ± 0.0	5.0 ± 0.9	99.9 ± 0.0
SD 3	41.9 ± 2.3	97.6 ± 0.1	36.5 ± 2.2	98.1 ± 0.1	9.7 ± 1.1	86.3 ± 0.1	11.2 ± 1.5	99.9 ± 0.0	12.5 ± 1.2	99.9 ± 0.0
Head circumference										
SD 1	10.7 ± 1.4	97.6 ± 0.1	4.1 ± 0.9	98.1 ± 0.1	0.3 ± 0.2	86.3 ± 0.1	0.4 ± 0.3	99.8 ± 0.0	0.5 ± 0.3	99.8 ± 0.0
SD 2	26.9 ± 2.1	97.7 ± 0.1	14.3 ± 1.6	98.1 ± 0.1	4.2 ± 0.9	86.3 ± 0.1	4.1 ± 1.0	99.8 ± 0.0	4.3 ± 0.9	99.8 ± 0.0
SD 3	38.7 ± 2.3	97.7 ± 0.1	25.4 ± 2.1	98.2 ± 0.1	10.7 ± 1.2	86.3 ± 0.1	12.9 ± 1.6	99.8 ± 0.0	11.3 ± 1.2	99.8 ± 0.0

∗ Linear equation of sex- and age-standardized z-score as a function of age (*Z*_*ij*_ = *β*_*0i*_ + *β*_*i*_*t*_*ij*_ + *ε*_*ij*_).

† Raw anthropometric measurement as a function of square root of age (*Y*_*ij*_ = *β*_*0i*_ + *β*_*i*_*t*_*ij*_^*½*^ + *ε*_*ij*_).

‡ Based on a random effects restricted cubic spline (with 5 knots) model.

## Supplementary Stata code for calculating jackknife residuals

/* (1) Set-up:

Note that the data should be in long format and the following numerical variables are used for this analysis:

- PartID: each child's unique identifier
- Age: age of child at time of measurement
- Length: length measurement of child
- LAZ: length-for-age z-score of child

It may be easier to rename the corresponding variables to match the variable names used in the subsequent code. */

/* (2) Generating New Variables */

sort PartID Age

generate SqrtAge=sqrt(Age) // square root of age

generate LAZ_Complete=1 if LAZ!=. // variable to indicate whether or not a measurement was completed at a given time point

generate Length_Complete=1 if Length!=.

generate LAZ_Residual=. // creating variable to store jackknife residual values from LAZ model

generate Length_Residual=.

by PartID: egen LAZ_Count=count(LAZ) // generating a count variable that indicates how many LAZ measurements are available per infant

by PartID: egen Length_Count=count(Length)

/* (3) Running Model for LAZ, Storing Residuals and Flagging Outliers*/

levels of PartID if LAZ_Count>3, local(levels) // storing all PartID values of children who have more than 3 measurements in local macro called 'levels'; note that this line of code needs to be run at the same time as the following loop

foreach id of local levels{ // looping through all PartIDs

regress LAZ Age if PartID=='id' // regressing LAZ on age for just one ID
predict Residual'id' if PartID=='id', rstudent // predicting jackknife residual
replace LAZ_Residual=Residual'id' if PartID=='id' // storing predicted jacknknife residual into LAZ_Residual variable
drop Residual'id'

}

/* (4) Running Model for Length, Storing Residuals and Flagging Outliers */

levels of PartID if Length_Count>3, local(levels)

foreach id of local levels{

regress Length SqrtAge if PartID=='id' // regressing length on squart root of age for just one ID
predict Residual'id' if PartID=='id', rstudent
replace Length_Residual=Residual'id' if PartID=='id'
drop Residual'id'

}

/* (5) Identifying Outliers */

* a. LAZ Model, Using Cutoff of +/- 4
generate LAZ_Outlier=0 if abs(LAZ_Residual)<4
  replace LAZ_Outlier=1 if abs(LAZ_Residual)>4 & LAZ_Residual!=.
  replace LAZ_Outlier=9 if LAZ_Residual==.
* b. Length Model, using Cutoff of +/- 4
generate Length_Outlier=0 if abs(Length_Residual)<4 // identifying outliers based on +/- cutoff
  replace Length_Outlier=1 if abs(Length_Residual)>4 & Length_Residual!=.
  replace Length_Outlier=9 if LAZ_Residual==.

/* Note that observations for which the jackknife residual could not be evaluated (i.e. participant had too few observations), the LAZ_Outlier variable will be indicated as '9', and manual review of these observations should be conducted */

/* (6) Identifying Implausible Values

Note: This assumes that any decrease in length from one time point to the next represents a pair of biologically implausible values, for which an error must have occurred in at least one of the two measurements */

/* a. Calculating Change in Length Between Successive Measurements */
sort PartID Length_Complete Age
by PartID: generate Length_Change=Length-Length[_n-1] // calculating change between successive measurements in the same infant
/* b. Identifying Pairs of Biologically Implausible Values */
by PartID: generate Length_BIVPair=_n if Length_Change[_n+1]<0
by PartID: replace Length_BIVPair=Length_BIVPair[_n-1] if Length_Change<0
/* c. Determining Incorrect Value Based on Jackknife Residuals from LAZ Model */
sort PartID Length_BIVPair
by PartID Length_BIVPair: egen LAZ_BIV_Max=max(abs(LAZ_Residual)) if Length_BIVPair!=.
generate LAZ_BIV=1 if abs(LAZ_BIV_Max)==abs(LAZ_Residual) & LAZ_BIV_Max!=.
/* d. Determining Incorrect Value Based on Jackknife Residuals from Length Model */
sort PartID Length_BIVPair
by PartID Length_BIVPair: egen Length_BIV_Max=max(abs(Length_Residual)) if Length_BIVPair!=.
generate Length_BIV=1 if abs(Length_BIV_Max)==abs(Length_Residual) & Length_BIV_Max!=.

## Appendix A Supplementary methods

### Generation of sex- and age-standardized z-scores for length, weight, and head circumference measurements

(1) Sex- and age-standardized z-scores for length, weight, and head circumference measurements were generated using the Intergrowth-21st Newborn Size standards for length, weight, and head circumference measurements taken within 48 hours of birth; (2) the Intergrowth-21st International Postnatal Growth Standards for Preterm Infants for length, weight, and head circumference measurements taken up to 64 weeks of postmenstrual age among preterm infants (born earlier than 37 weeks of gestation); and (3) the World Health Organization (WHO) Child Growth Standards for length, weight, and head circumference measurements among term infants and measurements taken after 64 weeks of postmenstrual age among preterm infants.

### Models for individual sex- and age-standardized z-scores and raw measurements for the jackknife residual method

Linear regression was used to fit a straight line through the individual's sex- and age-standardized z-score of the corresponding anthropometry measurement as a function of age:

(1) $$Z_{ij}=\beta_{0i}+\beta_{i}\cdot t_{ij}+\varepsilon_{ij}$$

where “*i*” denotes the *i*^th^ individual and “*j*” denotes the *j*^th^ time point.

For raw measurements, each individual's measurements were regressed on the square root of age (t^½^) to model a curvilinear relationship:

(2) $$Y_{ij}=\beta_{0i}+\beta_{i}\cdot t_{ij}^{1/2}+\varepsilon_{ij}$$

### Minimum number of measurements required per individual

In both the sex- and age-standardized z-score and raw measurement models, two parameters are being estimated: one for the intercept and one for age or square root of age. Because a line fit to two measurements is simply directly connecting one point to the other, a minimum of three measurements is required in order for there to be some variation (i.e., residual not equal to zero) around the fitted trajectory. In the case of calculating jackknife residuals, a fourth measurement is required since mean squared error is calculated with the *i*^*th*^ observation dropped. In general, (*k* + *2*) measurements per infant are required for the jackknife residual method, where *k* represents the number of parameters in the model.
